# Cross-Platform Transcriptomic Analysis of 40 Human and Rodent Skeletal Muscle Exerkines

**DOI:** 10.3390/muscles5010015

**Published:** 2026-02-13

**Authors:** Hash Brown Taha, Nathan Robbins, Firas-Shah Zoha, Shirley Zhu, Nandhana Vivek, Aleksander Bogoniewski

**Affiliations:** 1 School of Medicine, Washington University in St. Louis, St. Louis, MO 63110, USA; 2 School of Medicine, University of California San Francisco, San Francisco, CA 94143, USA; 3 Department of Biochemistry and Biomedical Sciences, McMaster University, Hamilton, ON L8S 4L8, Canada; 4 Department of Integrative Physiology, University of Colorado Boulder, Boulder, CO 80309, USA; 5 Department of Medical and Molecular Pharmacology, University of California Los Angeles, Los Angeles, CA 90095, USA

**Keywords:** exercise, exerkines, skeletal muscle, transcriptomics, meta-analysis

## Abstract

Animal and human studies show that exercise induces organism-wide molecular adaptations that are partly mediated by exerkines which are secreted factors that enable inter-organ communication between tissues such as skeletal muscle, adipose tissue, liver and the brain. However, the tissue-specific responsiveness of individual exerkines and how these responses differ across species, exercise conditions and sexes remain poorly understood. To address this gap, we systematically analyzed skeletal muscle transcriptomic responses of 40 exerkines using three publicly available datasets including MetaMEx, Extrameta and the MoTrPAC 6-month-old rat training dataset. We reviewed exerkine-specific regulation in humans, mice and rats across acute and chronic exercise and inactivity. We determined conserved, non-conserved, and discordant exerkines across species and whether they were dependent on exercise modality or sex. Our review reveals substantial heterogeneity in skeletal muscle transcriptomic exerkine regulation with only a small subset showing conserved changes across species. Additionally, a key limitation is that our analysis was limited to transcriptomic data and may not reflect protein-level abundance, secretion, or uptake by recipient tissues. Therefore, we highlight a need for multi species and multi condition approaches when selecting exerkines as biomarkers or surrogate therapeutic targets.

## 1. Introduction

Animal and human studies strongly support the role of exercise in causing organism-wide improvements in metabolism and health. “Walking is man’s best medicine” is a thousand-year-old quote from Hippocrates [1], illustrating that even with limited understanding of the mechanisms driving various diseases, physical activity and exercise have been recommended as a panacea. Studies have shown that the widespread benefits of exercise can be largely attributed to enhanced intra- and inter-organ communication through molecules termed ‘exerkines’ [2,3,4]. Indeed, re-infusing exercise-conditioned plasma into non-exercised mice confirms the presence of beneficial bioactive circulatory molecules by offering protection against age-related cognitive decline and enhancing memory [5,6].

Examples of these exerkines include apelin (APLN), fibroblast growth factor 21 (FGF21), interleukin-6 (IL-6), and brain-derived neurotrophic factor (BDNF) originating from various tissues such as adipose, skeletal muscle, liver, and the brain, exerting specific effects in the targeted tissue [2,3,7]. However, little is known about the specific molecular responses of exerkines within and across different tissues in multiple species (e.g., humans and rodents), or whether there are any intensity-related, time-dependent or sex-specific variations. This is largely attributed to the fact that conducting integrative systems-wide studies requires extensive funding, time and personnel.

Recently, we performed a global multi-omics analysis of 28 exerkines across 20 tissues in exercised 6-month-old rats using data from the Molecular Transducers of Physical Activity Consortium (MoTrPAC), and highlighted several intricate time-dependent, intensity-related, and sex-dimorphic responses. Importantly, despite skeletal muscle making up ~40% of our body weight and thought of as the primary source of exerkine communication in previous studies [2,8], we discovered that skeletal muscle (gastrocnemius and vastus lateralis) had little exerkine alterations.

To build on these findings and elucidate skeletal muscle-specific exerkine-responsiveness, we gathered a list of 38 known and 2 speculative exerkines, and analyzed their skeletal muscle gene expression using two meta-analyses and the MoTrPAC 6-month-old rat dataset. Since many studies do not directly quantify protein secretion across tissues, we leveraged skeletal muscle transcriptomic changes as a proxy for exercise-associated regulation of these candidate exerkines. We focused specifically on skeletal muscle because it is the most widely recognized and extensively studied source of exercise-induced circulating factors (“myokines”) and remains an important tissue linking physical activity to systemic adaptations. The two meta-analyses (MetaMEx [9] and Extrameta [10]) pooled gene expression data from published studies to query changes in skeletal muscle transcription responses due to exercise or inactivity, while the MoTrPAC is a global study that uses both human- and rat-based cohorts aiming to uncover the molecular mechanisms by which exercise benefits health [7]. In this study, we only leveraged their publicly available 6-month-old rat dataset.

The review’s goal is to pinpoint which skeletal muscle exerkines are most exercise-responsive, elucidate how they vary in multiple species (i.e., humans, mice and rats), and explore whether there are intensity-related, time-dependent, and/or sex-dimorphic variations.

## 2. Data Extraction and Analytical Framework

Meta-analytical data analyzed in this study are publicly available from MetaMEx [9] and Extrameta [10], while rat-specific data is publicly available from the MoTrPAC [7]. MetaMEx includes 66 transcriptomic studies for humans and 34 studies for mice, while Extrameta includes 43 studies for humans. Detailed and specific information on each dataset is included in their respective publication. Analyses in this review were made/conducted using https://metamex.serve.scilifelab.se/app/metamex for MetaMEx, https://extrameta.org/ for Extrameta, and the MoTrPACRatTraining6moData R package (version 2.0.0) in RStudio (version 4.2.2) for MoTrPAC.

We analyzed a list of 38 known exerkines and 2 speculative exerkines [2,5,6,11,12,13,14]. The exerkines include APLN, adiponectin (ADIPOQ), leptin (LEP), meteorin-like (METRNL), growth differentiation factor 15 (GDF15), insulin-like growth factor 1 (IGF-1), FGF21, carboxylesterase 2 and 2C (CES2), PPAR gamma coactivator 1-alpha (PPARGC1A, also known as PGC-1α), fibronectin type III domain-containing protein 5 (FNDC5)/irisin, cathepsin B (CTSB), IL-6, interleukin 7 (IL-7), interleukin 10 (IL-10), interleukin 13 (IL-13), interleukin 15 (IL-15), musclin/osteocrin (OSTN), myostatin (MSTN), follistatin (FST), follistatin like-1 (FSTL1), myonectin (ERFE/CTRP15), decorin (DCN), leukemia inhibitory factor (LIF), secreted protein acidic and rich in cysteine (SPARC), syndecan 4 (SDC4), transforming growth factor beta 1 (TGFβ1), transforming growth factor beta 2 (TGFβ2), angiopoietin 1 (ANGPT1), angiopoietin like-4 (ANGPTL4), fractalkine (CX3CL1), BDNF, neurturin 1 (NTN1), platelet factor 4 (PF4), klotho (KL), fibronectin-1 (FN1), glycosylphosphatidylinositol-specific phospholipase D1 (GPLD1), Clusterin (Clu), and fetuin-A (AHSG). We also included the speculative exerkines prosaposin (PSAP) and PSAP like 1 (PSAPL1) given that they are gaining attention as myokines and adipokines [15,16].

Significance for MetaMEx and Extrameta was determined based on whether the summary point’s error bar crossed the zero line [17]. Timeline-specific significance from MetaMEx was assessed based on a *p*-value cutoff < 0.05 and whether the logFC was meaningful. For the MoTrPAC, significance was determined using three consecutive conservative methods. *p*-adjusted values were used to evaluate overall significance. If the *p*-adjusted value was significant (<0.05), we used the sex-specific *p*-values to determine significance. In all cases, if the error bars crossed the zero value, significance was deemed negligible regardless of the *p*-value [18]. This was done to ensure that the specificity of the significance determination for exerkines is high. Acute exercise for MoTrPAC was defined as 1–2 weeks, while chronic exercise was defined as 4–8 weeks.

Review of each dataset and conclusions are all listed in Table 1.

## 3. Description and Rationale for Selection of Candidate Exerkines

APLN was identified as the ligand for the APJ receptor decades ago [19] and normally acts as a vasoactive metabolic peptide involved in vascular tone, cardiac function, angiogenesis, fluid balance, and insulin sensitivity [20]. Its role as an exerkine is known to be mostly related to skeletal muscle of exercised humans and rodents [21,22]. Several studies have demonstrated that apelin is capable of reversing age-related degenerative conditions such as sarcopenia [22,23].

ADIPOQ was first identified as a primarily adipose tissue protein that significantly impacts metabolic processes such as glucose regulation and fatty acid breakdown. Several studies have shown that it can have neurotrophic and neuroprotective effects, and plays significant roles in reducing inflammation, enhancing insulin sensitivity, and mitigating atherogenic processes [24,25]. Several studies have shown that exercise increases ADIPOQ levels in the circulation of humans [26,27,28], whereas rodent studies reported conflicting results [29,30,31].

LEP was first identified as the protein product of the obesity gene and is primarily secreted by adipose tissue, where it acts as a key regulator of energy balance by signaling nutritional status to the hypothalamus to control appetite and body weight [32]. In addition to its metabolic role, LEP influences neuroendocrine function, immune activity, and cardiovascular physiology, and has been linked to inflammatory and atherogenic pathways in states of leptin resistance [33]. In the context of exercise, circulating LEP is often reported to decrease in humans, particularly when training is accompanied by reductions in fat mass, although acute changes may be variable depending on energy balance, intensity, and duration [34].

METRNL is a secreted factor that has gained attention as a potential exerkine due to its links with exercise-related metabolic adaptation and immune signaling. It is thought to act as a communication signal between skeletal muscle and adipose tissue to support systemic energy homeostasis [35]. METRNL has important roles in regulating immune responses and metabolism, and is expressed in various tissues including skeletal muscle, BAT, and WAT, and is known to be exercise-responsive and cold-sensitive [36]. One of its main functions is facilitating BAT-to-WAT conversion and increasing adipose tissue thermogenesis in male rodents [37].

GDF15 is a stress-responsive cytokine within the TGF-β superfamily that has been implicated in the regulation of both inflammatory pathways and metabolic homeostasis. It is typically induced under conditions of cellular stress and disease, and can act centrally to influence appetite and whole-body energy balance. In peripheral tissues, GDF15 has also been linked to adipose remodeling, including promotion of WAT browning and increased thermogenic activity [38,39]. Two studies report that plasma GDF15 rises immediately following acute exercise in both male humans and rodent models [40,41]. In rodents, this acute response has additionally been associated with increased Gdf15 gene expression in the liver, heart, and skeletal muscle [41]. However, similar changes were not seen in studies using voluntary wheel running, suggesting that forced treadmill exercise conditions may be required to reliably elicit exercise-induced elevations in circulating and tissue GDF15 expression [40,41].

IGF-1 (also called somatomedin C) was originally identified as a circulating growth-promoting factor that mediates many of the anabolic effects of growth hormone. It is produced mainly by the liver (endocrine IGF-1), but it is also produced locally in tissues including skeletal muscle, where it supports muscle growth/repair, protein synthesis, and cell survival. IGF-1 is commonly discussed as an exercise-responsive factor because exercise and training can influence circulating IGF-1 and muscle IGF-1 signaling, though the direction and magnitude depend on training status, age, and metabolic context [42,43,44].

FGF21 is a liver-derived hormone with strong effects on glucose and lipid metabolism. It supports the body’s adaptation to stress and fasting by enhancing lipolysis, ketogenesis, and insulin sensitivity. FGF21 has also been linked to WAT browning and central regulation of energy expenditure, appetite, and body weight [45], and its activity can be influenced by the exerkine ADIPOQ [46]. Acute exercise has been reported to increase circulating FGF21 in humans, while rodent studies suggest increases in plasma and liver but not in skeletal muscle or WAT [47].

CES2 is a carboxylesterase enzyme best known for its role in drug and lipid metabolism, where it hydrolyzes ester-containing compounds and helps regulate triglyceride and cholesterol ester handling. It is expressed most strongly in the liver and intestine (and can be present in other metabolic tissues), making it more of a metabolic enzyme marker than a classic secreted “hormone-type” exerkine [48]. However, a recent study showed that CES2 can be secreted as an exercise-responsive extracellular enzyme, with particularly strong induction from the liver after one week of treadmill running. The authors also found that lactate can stimulate CES2 secretion from hepatocytes, and engineered soluble CES2 was sufficient to produce anti-obesity, anti-diabetic, and endurance-enhancing effects in mice [14].

PPARGC1A encodes PGC-1α, a master transcriptional coactivator that was originally identified as a regulator of energy metabolism through its interaction with nuclear receptors. In normal physiology, PPARGC1A drives mitochondrial biogenesis, oxidative phosphorylation capacity, and fatty acid oxidation, making it a central controller of endurance-type metabolic programs. It is strongly expressed and inducible in skeletal muscle, and it is widely considered a key exercise-responsive mediator because endurance exercise robustly increases PPARGC1A expression in muscle and activates downstream pathways that support metabolic adaptation. While PPARGC1A itself is not a secreted exerkine, it is a major upstream regulator that controls multiple exercise-linked secreted factors (exerkines) and muscle remodeling responses [49,50,51].

FNDC5/irisin has gained major attention as a putative exerkine because of its proposed roles in metabolic regulation and overall health. Irisin is generated by proteolytic cleavage of the type I membrane protein FNDC5 and is thought to be produced mainly by skeletal muscle in response to exercise. In both male and female mice given free access to running wheels for three weeks, as well as in male humans completing 10 weeks of aerobic training, increased FNDC5 expression was observed in the quadriceps along with elevated circulating irisin levels [52]. Since these initial reports, multiple studies have described exercise-associated increases in FNDC5 in humans and rodent models [53,54,55], although the reliability and interpretation of some findings remain controversial [56,57].

CTSB is a lysosomal cysteine protease that plays a key role in intracellular protein degradation and turnover. Its classification as an exerkine remains debated, as studies in young male humans show that acute exercise does not raise plasma CTSB levels [58], whereas other work in young male and female humans, rhesus monkeys, and mice with voluntary wheel access suggests that chronic exercise is associated with increased circulating CTSB [59,60]. In addition, in young male and female mice allowed voluntary wheel running for 30 days, *Ctsb* expression increased in gastrocnemius skeletal muscle [60].

PSAP is a multifunctional lysosomal protein first described in 1988 that serves as the precursor for saposins A–D, which are required for normal lysosomal lipid degradation [61]. PSAP has also been studied for neuroprotective and therapeutic potential in neurologic disease contexts. Although PSAP has been speculated to be exercise-responsive because its levels increase in skeletal muscle, adipose tissue, and their extracellular fluids under PGC1α overexpression (an established “exercise-like” muscle state) and after cold exposure in adipose tissue, available evidence suggests PSAP is most likely not an exerkine. Specifically, proteomic analyses did not detect PSAP as a differentially altered protein in skeletal muscle extracellular fluid from mice exposed to an acute treadmill-running protocol compared with sedentary controls [15], and an aptamer-based proteomics study reported that acute exercise decreases PSAP in the plasma of older male humans [61]. While TFEB overexpression studies aiming to mimic an exercise-like state have reported higher PSAP levels in select tissues [62], these findings are limited by small sample sizes and methodological constraints. Overall, the current body of evidence supports the interpretation that PSAP may respond to metabolic stress in certain contexts but is unlikely to be a true exerkine, nor a consistent skeletal muscle-derived exercise-responsive molecule or mediator of exercise adaptation [61].

IL-6 is a pleiotropic cytokine with broad functions in immunity, inflammation, hematopoiesis, skeletal muscle and bone maintenance, brain activity, and whole-body metabolism [63]. In 2000, IL-6 was identified as a skeletal muscle-derived exerkine in male humans after an acute bout of one-legged knee-extension exercise [64], and many subsequent studies have shown increases in circulating IL-6 with both short- and longer-term exercise in humans and rodents [65,66]. IL-7 is a cytokine involved in inflammatory signaling that is often discussed as an exerkine, with proposed roles during physical activity in shaping inflammatory responses and supporting muscle repair and recovery, although its exercise responsiveness across humans and rodents remains an active area of investigation [67,68]. IL-10 is a key anti-inflammatory cytokine that is commonly elevated during exercise in humans [69], with less consistent findings in rodents [70], and its induction is frequently linked to IL-6 signaling, consistent with a feedback mechanism in which IL-6 promotes IL-10 release to limit inflammation and support post-exercise recovery [71]. IL-13, a T helper 2 cytokine, contributes to anti-inflammatory immune responses and macrophage polarization in WAT [72], and has also been reported to behave as an exerkine by increasing in circulation after several weeks of aerobic training in humans and treadmill exercise in rodents; importantly, IL-13 deficiency has been associated with reduced exercise capacity and weaker metabolic benefits in rodent models [73]. IL-15 supports immune regulation by promoting the development and function of natural killer cells as well as T and B lymphocytes, but exercise effects on circulating IL-15 are inconsistent, with many studies showing no change after acute or chronic exercise in humans while others report increases only in specific settings (e.g., acute bouts or female-specific responses), with similarly mixed findings in rodents [74,75].

OSTN was first described as a factor produced by skeletal muscle [76]. Its expression and protein levels have been reported to increase in the skeletal muscle of young male and female rodents after 5 days of treadmill training, with corresponding elevations also observed in circulation [77]. In humans, acute exercise has likewise been shown to raise circulating musclin levels in young inactive males during the workout and for up to 120 min afterward [78].

MSTN, also referred to as GDF-8, is a TGFβ family protein that acts as an endogenous inhibitor of skeletal muscle growth, helping prevent excessive hypertrophy. In the context of exercise, myostatin levels are often reported to decrease to support muscle adaptation and growth, although some studies suggest a modest early rise may occur during intense activity as a short-term regulatory signal to constrain muscle expansion within safe limits [2,79].

FST and FSTL1 are secreted proteins that have been studied in the context of skeletal muscle remodeling, cardiometabolic regulation, and exercise responsiveness. FST is best known as a high-affinity binding protein that neutralizes myostatin (GDF-8) and activins, thereby reducing inhibitory signals on muscle growth and supporting muscle repair and hypertrophy; because of this, it is often discussed as an exercise-responsive myokine that may increase with training and contribute to adaptation by shifting the balance toward anabolic signaling. FSTL1, while structurally related in name, is functionally distinct and is involved in inflammatory signaling, tissue remodeling, and cardiovascular biology, with evidence supporting roles in endothelial function and cardiac protection; it has also been reported to be exercise-responsive in some contexts and is frequently framed as a candidate exerkine linking skeletal muscle activity to systemic vascular and metabolic effects [80,81,82,83].

Myonectin (CTRP15), also known as ERFE, is a secreted factor originally described as a muscle-associated circulating protein linked to metabolic regulation. It has been studied for its role in coordinating nutrient handling, particularly by promoting lipid uptake and supporting systemic energy homeostasis, which is why it is frequently discussed as a candidate myokine/exerkine. In addition, ERFE has been connected to erythropoiesis-related signaling and iron regulation, highlighting that this molecule may have context-dependent endocrine effects beyond metabolism, and its circulating levels and responsiveness can vary depending on physiologic state and experimental conditions [84,85,86,87].

DCN is a small leucine-rich proteoglycan found in the extracellular matrix that was originally characterized for its role in organizing collagen fibrils and maintaining connective tissue structure. Beyond its structural function, DCN influences cell signaling and tissue remodeling, including modulation of growth factor activity such as TGFβ, and it has been linked to muscle repair and fibrosis control. In the context of exercise, DCN is often discussed as a candidate myokine/exerkine because its expression can increase in skeletal muscle with training and it may contribute to adaptive remodeling by counteracting pro-fibrotic pathways and supporting healthy muscle extracellular matrix turnover [88,89,90,91,92].

LIF is a pleiotropic cytokine in the IL-6 family that regulates a broad range of processes including cell growth, differentiation, and survival. It contributes to normal physiology through roles in immune signaling, neuronal development, and skeletal muscle repair, and is often induced by stress or injury such as exercise. During exercise, LIF can support regeneration by activating pathways such as JAK/STAT in muscle and other tissues [93], and its short-lived elevation suggests a role in tissue adaptation, although responses vary depending on exercise type, intensity, and duration [94,95]. LIF has also been widely described as cardioprotective, with evidence supporting functions in cardiac repair and protection against cellular injury [96].

SPARC participates in multiple physiological processes such as wound repair, bone mineralization, angiogenesis, and extracellular matrix remodeling. It has also been reported to be exercise-responsive, with studies showing increased secretion following exercise in both humans and rodent models [97,98].

SDC4 is a transmembrane heparan sulfate proteoglycan that regulates cellular behaviors such as migration, proliferation, and differentiation through interactions with extracellular matrix components. It has been implicated in skeletal muscle remodeling by supporting signaling pathways involved in muscle repair and growth [99,100]. Exercise has been reported to increase circulating SDC4 in young male humans and in rodent models [101], and it has also been proposed that SDC4 may function as a hepatokine [101].

TGFβ1 and TGFβ2 are members of the TGFβ superfamily and act as major regulators of cellular growth, proliferation, differentiation, and apoptosis. TGFβ1 is well recognized for its roles in immune regulation, angiogenesis, and particularly fibrosis, and is often viewed as a key pro-fibrotic mediator in many disease settings. TGFβ2 shares overlapping functions but is more prominently associated with specific tissues such as the brain, heart, and eye, where it contributes to embryonic development and maintenance of tissue homeostasis [102]. Both TGFβ1 and TGFβ2 have been reported to increase following acute and/or chronic exercise in humans and rodent models [103,104,105,106]. TGFβ1 is also relevant to skeletal muscle biology, with exercise shown to elevate its expression in skeletal muscle in both humans and rodents [103,104,105]. In contrast, TGFβ2 has been proposed to function as an exerkine derived primarily from WAT [106].

ANGPT1 is a growth factor that plays a key role in vascular stabilization and angiogenesis by activating the TIE2 receptor on endothelial cells to strengthen vascular integrity and limit permeability. It has been reported to increase after acute exercise in both humans and rodents [107,108,109], while findings following chronic exercise appear more variable [108,109]. ANGPTL4, a related angiopoietin-like protein, is also involved in vascular and metabolic regulation, particularly lipid handling, and has likewise been described as exercise-responsive in several contexts, reflecting its role in coordinating vascular and metabolic adaptations to physical activity [110,111,112].

Fractalkine (CX3CL1) is a unique molecule that acts both as a chemokine and an adhesion signal. Studies suggest that exercise can have inconsistent effects on circulating fractalkine levels in humans [113,114,115] and rodents [116]. It has also been described as an exerkine released from skeletal muscle with downstream actions on immune cells [114]. Although relatively few rodent studies have examined how fractalkine protein levels and gene expression change across multiple tissues with exercise, our analysis found exercise-related differences in fractalkine protein abundance in eight tissues (cortex, hippocampus, heart, kidney, adrenal gland, spleen, lungs, and BAT) and changes in gene expression in four tissues (heart, adrenal gland, lungs, and BAT).

BDNF is a central neurotrophic factor with established roles in neuronal survival, growth, differentiation, and cognitive processes such as learning and memory [117]. In humans, acute exercise has been reported to raise circulating BDNF levels in males [58,118], while findings from chronic exercise interventions remain inconsistent [119]. In rodent models, both acute and chronic exercise have generally been reported to have little effect on plasma BDNF [120,121]. In contrast, chronic exercise robustly increases BDNF within the brain, particularly in the hippocampus [122], which aligns with meta-analytic evidence showing elevated brain BDNF levels in exercised male and female rodents [123].

NTN1 is a secreted guidance cue best known for its roles in neuronal development, where it directs axon growth and cell migration, but it also has important functions outside the nervous system in vascular biology, inflammation, and tissue remodeling. NTN1 is expressed across multiple tissues (including brain, immune cells, endothelium, and metabolic tissues) and has been implicated in regulating leukocyte trafficking and endothelial behavior, which makes it a plausible candidate for exercise-related signaling. In the context of exercise, NTN1 has been discussed as a potential exerkine-like factor, mainly because exercise can influence inflammatory and vascular pathways that NTN1 participates in, although the strength of evidence for NTN1 as a consistent exercise-responsive circulating exerkine is still emerging and appears to be context dependent [124,125,126].

PF4 is a platelet-derived chemokine classically linked to coagulation, but more recent work has also connected it to aging-related processes and neurological health [5,11,127,128]. In addition, circulating PF4 has been reported to increase after an acute exercise bout in rodent models [12,129].

FN1 encodes fibronectin, a major extracellular matrix glycoprotein that was originally characterized for its role in cell adhesion and tissue architecture, and is essential for processes such as wound repair, cell migration, and extracellular matrix remodeling. FN1 is produced by multiple tissues (including fibroblasts, endothelial cells, and liver) and can also be detected in circulation, where it reflects ongoing tissue remodeling and vascular or connective tissue dynamics. In the context of exercise, FN1 is often discussed as an exercise-responsive extracellular matrix-associated molecule because physical activity induces mechanical and inflammatory signaling that drives remodeling in skeletal muscle, vasculature, and other organs, which can alter FN1 expression and/or circulating levels depending on the tissue and training paradigm [130,131].

GPLD1 is an enzyme that modulates cell-surface signaling by cleaving glycosylphosphatidylinositol anchors, thereby influencing cell communication and downstream signaling pathways. Acute exercise has been reported to increase circulating GPLD1 in both humans and rodents, and these elevations have been proposed to contribute to neuroprotective effects [5].

Clusterin is a secreted glycoprotein involved in processes such as lipid transport, tissue remodeling, and regulation of cell survival and death, and it is often induced under conditions of tissue stress or injury. More recently, circulating clusterin has been reported to increase in male rodents given free access to a running wheel, with evidence suggesting associated neuroprotective and disease-modifying effects on cognitive decline [6].

Fetuin-A is a liver-derived circulating glycoprotein that was originally characterized for its roles in regulating mineral metabolism, including inhibiting ectopic calcification, and it also participates in metabolic and inflammatory signaling. In cardiometabolic contexts, Fetuin-A has been linked to insulin resistance and altered lipid handling, and circulating levels are often studied as a biomarker of metabolic health. In the setting of exercise, Fetuin-A is commonly discussed as an exercise-responsive hepatokine, with reported changes in circulation depending on training status, baseline metabolic state, and whether exercise improves insulin sensitivity or reduces adiposity [132,133].

## 4. Cross-Platform Analysis of Exerkines Based on Species and Exercise Modality

**Figure 1:**
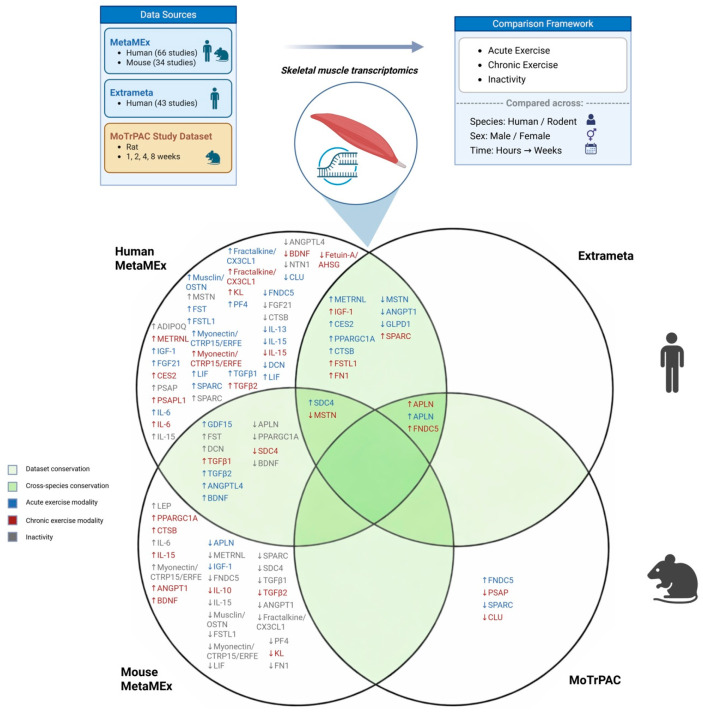
Cross-platform and cross-species overlap of exercise-responsive skeletal muscle exerkine transcripts. Schematic overview of the three transcriptomic resources used in this study (MetaMEx: human and mouse; Extrameta: human; MoTrPAC: rat) and the comparison framework across acute exercise, chronic exercise, and inactivity, spanning both sexes and time points from hours to weeks. The Venn diagram summarizes shared and dataset-specific regulation of skeletal muscle exerkine-associated transcripts across Human MetaMEx, Mouse MetaMEx, Extrameta, and MoTrPAC. Overlapping regions indicate exerkines with concordant regulation across datasets and/or species, whereas non-overlapping regions represent context- or dataset-specific responses. Gene labels are color-coded by condition (acute exercise, chronic exercise, inactivity) and arrows denote the direction of change (↑ upregulated, ↓ downregulated) in skeletal muscle.

Below we provide a brief description and patterns of the analysis. The reader should refer to Table 1 for more detailed and nuanced comparisons, or Figure 1 for a graphical summary. Throughout the below sections, exerkines are discussed at the transcript level and gene symbols are presented in full uppercase (non-italicized) to improve readability.

### 4.1. Exerkines Altered in Humans and Rodents Due to Acute Exercise

Several exerkines show consistent alterations in response to acute exercise across species. In humans, acute exercise increases APLN, GDF15, IGF-1, PPARGC1A, SDC4, TGFB2, ANGPTL4, and BDNF. In rodents, findings are more variable: APLN decreases in MetaMEx (primarily in males) but increases in MoTrPAC females at 1–2 weeks; IGF-1 appears to decrease in rodents (MetaMEx only) despite increasing in humans (HIIT exercise only); and GDF15, PPARGC1A, SDC4, TGFB2, and ANGPTL4 generally increase. BDNF is elevated in humans and rodents (MetaMEx) following acute exercise. These patterns highlight both conserved and species-specific exerkine responses to exercise.

### 4.2. Exerkines Altered in Humans and Rodents Due to Chronic Exercise

During chronic exercise, only a limited number of skeletal muscle exerkines showed convergent regulation across humans and rodents. APLN and FNDC5 consistently increased in both species, with rodent findings supported by both MetaMEx and MoTrPAC, indicating robust cross species conservation. MSTN also showed concordant decreases in humans and rodents, consistent with its known suppression by training. In contrast, several exerkines displayed opposing regulation between species, including IL15 (decreased in humans, increased in rodents), TGFB2 (increased in humans, decreased in rodents), BDNF (decreased in humans, increased in rodents), and KL (increased in humans, decreased in rodents). Other molecules such as PPARGC1A, SDC4, and TGFB1 showed clear regulation in humans but limited or MetaMEx only evidence in rodents, highlighting species-specific transcriptional responses. Interestingly, two exerkines showed contrasting results in humans and rodents using MoTrPAC. SPARC and myonectin increased in humans and decreased in rodents. Collectively, aside from APLN and FNDC5, most chronic exercise-induced skeletal muscle exerkine changes lacked consistent cross species convergence.

### 4.3. Exerkines Altered in Humans and Mice Due to Inactivity

Inactivity leads to a coordinated suppression of several exercise-responsive exerkines across species. Both humans and rodents show decreased APLN, PPARGC1A, and BDNF, alongside increases in FST and DCN. Some molecules display species-specific divergence: IL-15 increases in humans but decreases in rodents, while SPARC increases in humans but decreases in rodents. These patterns highlight shared inactivity-induced molecular signatures as well as important species-dependent regulatory differences.

### 4.4. Exerkines Altered Only in Humans Due to Acute Exercise

Several exerkines exhibit acute exercise responsiveness uniquely in humans, with no corresponding alterations detected in rodents. Human-specific increases were observed for METRNL, FGF21, CES2, CTSB, IL-6, musclin/OSTN, FST, FSTL1, myonectin, LIF, SPARC, and TGFB1, while METRNL and SPARC showed partial support from MoTrPAC (e.g., SPARC at 2 weeks). Additional human-specific decreases were noted for FNDC5, IL-13, IL-15, DCN, ANGPT1, GPLD1, and CLU. Some molecules demonstrated mixed or context-dependent effects, including fractalkine and PF4. These findings highlight that several acute exerkine responses may be human-exclusive or substantially more pronounced in humans than in rodents, emphasizing important species differences in early molecular responses to exercise.

### 4.5. Exerkines Altered Only in Humans Due to Chronic Exercise

A subset of exerkines demonstrated chronic exercise responsiveness exclusively in humans, with no corresponding alterations observed in rodents. Human-only increases included METRNL, IGF-1, IL-6 (notably reported only in a single male cohort), FSTL1, fractalkine, and fibronectin (FN1). Additionally, fetuin-A showed a human-specific decrease. These findings indicate that several chronic exerkine adaptations may be uniquely human or substantially more prominent in humans than in rodents, underscoring important species-specific differences in longer-term molecular responses to exercise.

### 4.6. Exerkines Altered Only in Rodents Due to Acute Exercise

No exerkines showed acute exercise-specific alterations exclusively in rodents, indicating that all acute rodent responses overlapped with human findings or were not uniquely rodent-specific.

### 4.7. Exerkines Altered Only in Rodents Due to Chronic Exercise

Chronic exercise produced several rodent-specific exerkine alterations that were not observed in human cohorts. These included a decrease in IL-10, an increase in CTSB (restricted to males), a decrease in PSAP (observed at 4 weeks in females and at 8 weeks in both sexes in MoTrPAC), and an increase in ANGPT1.

### 4.8. Exerkines Altered Only in Humans Due to Inactivity

Inactivity produced several human-specific exerkine alterations not observed in rodent models. These included an increase in ADIPOQ (female only), PSAP, and MSTN, and decrease in FGF21, CTSB, ANGPTL4, and NTN1.

### 4.9. Exerkines Altered Only in Rodents Due to Inactivity

Rodent-specific analyses revealed a broad suppression of exerkine signaling during inactivity, highlighting a coordinated downregulation across multiple molecular pathways. Several key exerkines consistently decreased, including METRNL, FNDC5, Musclin/OSTN, FSTL1, LIF, SDC4, TGFβ1, ANGPT1, Fractalkine and FN1, indicating reduced myokine, adipokine, and matrix-associated activity in the inactive state. Additional decreases were observed in myonectin with mixed sex-specific responses, and PF4, while IL-6 uniquely increased under inactivity. Notably, leptin increased only in females, suggesting a sex-dependent metabolic response. Overall, these findings show that rodents exhibit a more uniform and widespread suppression of exerkine pathways during inactivity compared with humans, underscoring species-specific differences in how sedentary states modulate molecular signaling.

### 4.10. Exerkines Not Altered/Low Evidence

A small group of exerkines showed no consistent or biologically meaningful changes across exercise or inactivity conditions, including IL-7, IL-10, and PSAPL1, the latter having only a single low-evidence report of increased expression during chronic exercise in MetaMEx.

## 5. Discussion

In this cross-platform analysis, we demonstrate that skeletal muscle exerkine regulation is highly heterogeneous and strongly dependent on species, exercise duration, and physiological context. Only a limited subset of skeletal muscle exerkines showed concordant regulation across species and independent datasets, while most demonstrated species- and context-dependent behavior (Table 1, Figure 1).

**Table 1:** Skeletal muscle-specific exercise responsiveness. MetaMEx data is obtained from https://metamex.serve.scilifelab.se/app/metamex, Extrameta from https://extrameta.org/, and MoTRPAC data from MoTrPACRatTraining6moData R package (version 2.0.0) in RStudio (version 4.2.2). Exact definition metrics for each condition including aerobic, resistance, and high-intensity interval training (HIIT), inactivity, long-term and 1, 2, 4, and 8 weeks of training can be obtained from the respective studies. Abbreviations: M—male, F—female, B—both M and F separately, C—combined M and F, **APLN**—apelin, **ADIPOQ**—adiponectin, **LEP**—leptin, **METRNL**—meteorin-like, **GDF15**—growth differentiation factor 15, **IGF-1**—insulin-like growth factor 1, **FGF21**—fibroblast growth factor 21, **CES2**—carboxylesterase 2, **PPARGC1A**—**PPAR** gamma coactivator 1-alpha, **FNDC5**—fibronectin type III domain-containing protein 5, **CTSB**—cathepsin B, **PSAP**—prosaposin, **PSAPL1**—prosaposin-like 1, **IL-6**—interleukin 6, **IL-7**—interleukin 7, **IL-10**—interleukin 10, **IL-13**—interleukin 13, **IL-15**—interleukin 15, **Musclin/OSTN**—musclin/osteocrin, **MSTN**—myostatin, **FST**—follistatin, **FSTL1**—follistatin-like-1, **Myonectin/CTRP15/ERFE**—myonectin/C1q TNF related protein 15/erythroferrone, **Decorin**—decorin, **LIF**—leukemia inhibitory factor, **SPARC**—secreted protein acidic and rich in cysteine, **SDC4**—syndecan 4, **TGFβ1**—transforming growth factor beta 1, **TGFβ2**—transforming growth factor beta 2, **ANGPT1**—angiopoietin 1, **ANGPTL4**—angiopoietin-like 4, **Fractalkine/CX3CL1**—fractalkine/C-X3-C motif chemokine ligand 1, **BDNF**—brain-derived neurotrophic factor, **NTN1**—netrin 1, **PF4**—platelet factor 4, **KL**—klotho, **FN1**—fibronectin 1, **GPLD1**—glycosylphosphatidylinositol-specific phospholipase D1, **CLU**—clusterin, **Fetuin-A/AHSG**—fetuin-A/alpha-2-HS-glycoprotein.

Species	Human										Rodents						
Exerkine	MetaMEx Human								Extrameta		Metamex (Mouse)			MoTRPAC (Rat)			
	Acute				Chronic			Inactivity	Acute	Chronic	Acute	Chronic	Inactivity	1 Week	2 Week	4 Week	8 Week
	Aerobic	Resistance	HIIT	Timeline	Aerobic	Resistance	HIIT	NA									
APLN	↑ (M, C)	↑ (M, C)	↑ (M, C)	↑ 0–1, 2–3 and 4–6 h	↑ (M, C)	↑ (F, C)	↑ (C only M data and undefined included)	↓ (B, C)	↑	↑	↓ (M, C)	←→ (M)	↓ (M, C)	↑ (F) VL	↑ (F) VL	↑ (F) VL	↑ (F) VL
ADIPOQ	←→ (B, C)	←→ (B, C)	←→ (B, C)	↓ 0–1 and 2–3 h	←→ (B, C)	←→ (B, C)	←→ (only M data included)	↑ (F)	NA	←→	←→ (B, C)	←→ (only M data included)	←→ (B, C)	←→ (B)	←→ (B)	←→ (B)	←→ (B)
LEP	←→ (B, C)	←→ (B, C)	←→ (B, C)	NA	←→ (B, C)	←→ (B, C)	←→ (M, n = 1)	←→ (B, C)	NA	NA	←→ (B, C)	←→ (only M data included)	↑ (F)	←→ (B)	←→ (B)	←→ (B)	←→ (B)
METRNL	↑ (B, C)	↑ (B, C)	←→ (B, C)	↑ 0–1, 2–3 and 4–6 h	↑ (M, C)	←→ (B, C)	←→ (only M data)	←→ (B, C)	↑	←→	←→ (only M data)	←→ (only M data)	↓ (M, C)	←→ (B)	←→ (B)	←→ (B)	←→ (B)
GDF15	↑ (B, C)	←→ (B, C)	←→ (B, C)	NA	←→ (B, C)	←→ (B, C)	←→ (only M data)	←→ (B, C)	NA	NA	↑ (C)	←→ (M)	←→ (B, C)	NA	NA	NA	NA
IGF-1	←→ (B, C)	←→ (B, C)	↑ (M, C)	←→ (0–1 h −0.03 logFC. 2–3 0.013 log FC)	↑ (B, C)	↑ (B, C)	↑ (M data and undefined)	←→ (B, C)	←→	↑	↓ (M, C)	←→ (M)	←→ (B, C)	←→ (B)	←→ (B)	←→ (B)	←→ (B)
FGF21	↑ (F)	←→ (B, C)	←→ (B, C)	NA	←→ (B, C)	←→ (B, C)	←→ (M, n = 1)	↓ (M, C)	←→	←→	←→ (B, C)	←→ (M)	←→ (B, C)	NA			
CES2	↑ (B, C)	↑ (B, C)	↑ (M, C)	↑ 2–3, 4–6, 24 and 48 h	←→ (B, C)	←→ (B, C)	←→ (only M data)	←→ (B, C)	↑	←→	NA						
PPARGC1A	↑ (B, C)	↑ (B, C)	↑ (M, C)	↑ 0–1, 2–3 and 4–6 h	↑ (M, C)	←→ (B, C)	←→ (M)	↓ (B, C)	↑	←→	↑ (B, C)	↑ (M)	↓ (M, C)	←→ (B)	←→ (B)	←→ (B)	←→ (B)
FNDC5	↓ (M, C)	↓ (M, C)	←→ (B, C)	↓ 24 h	←→ (B, C)	←→ (B, C)	←→ (M)	←→ (B, C)	←→	↑	←→ (B, C)	↑ (M)	↓ (F (n = 1), C)	↑ (F) VL	↑ (F) VL	↑ (F) VL	↑ (F) VL
CTSB	↑ (M, C)	↑ (B, C)	↑ (M, C)	↑ 2–3, 4–6, 24 and 48 h	←→ (B, C)	←→ (B, C)	←→ (M)	↓ (M, C)	↑	←→	←→ (B, C)	↑ (M)	←→ (B, C)	←→ (B)	←→ (B)	←→ (B)	←→ (B)
PSAP	←→ (B, C)	←→ (B, C)	←→ (B, C)	↓ 0–1 and 2–3 h ↑ 48 h	←→ (B, C)	←→ (B, C)	←→ (only M data)	↑ (B, C)	←→	←→	←→ (B, C)	←→ (M)	←→ (B, C)	←→ (B)	←→ (B)	↓ (F) VL	↓ (B) VL
PSAPL1	←→ (only M data)	←→ (B, C)	←→ (B, C)	NA	←→ (B, C)	←→ (B, C)	↑ (M, n = 1)	←→ (B, C)	NA	NA	←→ (B, C)	←→ (M)	←→ (B, C)	NA	NA	NA	NA
IL-6	↑ (B, C)	↑ (M, C)	←→ (B, C)	NA	←→ (B, C)	←→ (B, C)	↑ (M, n = 1)	←→ (B, C)	NA	NA	←→ (B, C)	←→ (M)	↑ (C)	NA	NA	NA	NA
IL-7	←→ (only M data)	←→ (B, C)	←→ (B, C)	NA	←→ (B, C	←→ (B, C)	←→ (M, n = 1)	←→ (B, C)	NA	NA	←→ (B, C)	←→ (M)	←→ (B, C)	NA	NA	NA	NA
IL-10	←→ (only M data)	←→ (B, C)	←→ (B, C)	NA	←→ (B, C)	←→ (B, C)	←→ (M, n = 1)	←→ (B, C)	NA	NA	←→ (B, C)	↓ (M)	←→ (B, C)	NA	NA	NA	NA
IL-13	←→ (only M data)	←→ (B, C)	↓ (M, C)	←→	←→ (B, C)	←→ (B, C)	←→ (M, n = 1)	←→ (B, C)	NA	NA	←→ (B, C)	←→ (M)	←→ (B, C)	NA	NA	NA	NA
IL-15	↓ (M, C)	↓ (B, C)	↓ (B, C)	↓ 0–1 and 2–3 h ↑ 4–6, 24 and 48 h	↓ (M, C)	↓ (C)	←→ (M)	↑ (M, C)	←→	←→	←→ (B, C)	↑ (M)	↓ (M, C)	←→ (B)	←→ (B)	←→ (B)	←→ (B)
Musclin/OSTN	←→ (B, C)	←→ (B, C)	↑ (M, C)	NA	←→ (B, C)	←→ (B, Ct)	←→ (only M data and undefined)	←→ (B, C)	NA	NA	←→ (B, C)	←→ (M)	↓ (F, C)	←→ (B)	←→ (B)	←→ (B)	←→ (B)
MSTN	↓ (B, C)	↓ (B, C)	↓ (F)	↓ 2–3, 4–6, 24 and 48 h	↓ (M, C)	↓ (C)	↓ (only M data)	↑ (B, C)	↓	↓	←→ (B, C)	↓ (M)	←→ (B, C)	←→ (B)	←→ (B)	←→ (B)	←→ (B)
FST	←→ (B, C)	←→ (B, C)	↑ (M, C)	↑ 0–1 and 2–3 h	←→ (B, C)	←→ (B, C)	←→ (only M data)	↑ (B, C)	NA	←→	←→ (B, C)	←→ (M)	↑ (C)	←→ (B)	←→ (B)	←→ (B)	←→ (B)
FSTL1	↑ (M, C)	←→ (B, C)	↑ (M, C)	↑ 2–3 (logfC for 0–1 is 0.0017)	↑ (M, C)	↑ (C)	←→ (only M data) Remove undefined	←→ (B, C)	←→	↑	←→ (B, C)	←→ (M)	↓ (F, C)	←→ (B)	←→ (B)	←→ (B)	←→ (B)
Myonectin/CTRP15/ERFE	↑ (B, C)	↑ (B, C)	←→ (B, C)	NA	↑ (M)	←→ (B, C)	←→ (only M data)	←→ (B, C)	NA	←→	←→ (B, C)	←→ (M)	↓ (M, n = 1) ↑ (F, n = 1)	←→ (B)	←→ (B)	←→ (B)	↓ (B) G
Decorin (DCN)	←→ (B, C)	↓ (M, C)	←→ (B, C)	←→	←→ (B, C)	←→ (B, C)	←→ (only M data)	↑ (M)	←→	←→	←→ (B, C)	←→ (M)	↑ (M, C)	←→ (B)	←→ (B)	←→ (B)	←→ (B)
LIF	↑ (B, C)	←→ (B, C)	←→ (B, C)	↓ 0–1, 2–3 and 48 h	←→ (B, C)	←→ (B, C)	←→ (M, n = 1)	←→ (B, C)	←→	←→	←→ (B, C)	←→ (M)	↓ (M)	NA	NA	NA	NA
SPARC	←→ (B, C)	←→ (B, C)	↑ (C)	←→	↑ (B, C)	↑ (B, C)	↑ (only M data)	↑ (C)	←→	↑	←→ (B, C)	←→ (only M data)	↓ (M, C)	←→ (B)	↓ (M) G	←→ (B)	↓ (M) G
SDC4	↑ (B, C)	↑ (B, C)	↑ (B, C)	↑ 0–1, 2–3 and 4–6 h	←→ (B, C)	↓ (B, C)	↓ (only M data)	←→ (B, C)	↑	←→	↑ (M, C)	↓ (only M data available)	↓ (C)	←→ (B)	←→ (B)	←→ (B)	←→ (B)
TGFβ1	↑ (B, C)	↑ (F)	←→ (B, C)	←→ (logFC is -0.02 for 2–3 h)	↑ (M, C)	↑ (B, C)	↑ (only M data available)	←→ (B, C)	NA	←→	←→ (B, C)	↑ (only M data)	↓ (F, C)	←→ (B)	←→ (B)	←→ (B)	←→ (B)
TGFβ2	↑ (M, C)	↑ (B, C)	↑ (M, C)	↑ 2–3 and 4–6 h ↓ 24 h	↑ (M)	↑ (C) Add undefined	←→ (M)	←→ (B, C)	NA	NA	↑ (M, C)	↓ (only M data)	←→ (B, C)	←→ (B)	←→ (B)	←→ (B)	←→ (B)
ANGPT1	↓ (B, C)	↓ (B, C)	↓ (F)	↓ 2–3 and 4–6 h ↑ 24 h	←→ (B, C)	←→ (B, C)	←→ (only M data)	←→ (B, C)	↓	←→	←→ (B, C)	↑ (only M data)	↓ (M)	←→ (B)	←→ (B)	←→ (B)	←→ (B)
ANGPTL4	↑ (B, C)	↑ (F, C)	↑ (M)	↑ 0–1, 2–3 and 4–6 h	←→ (B, C)	←→ (B, C)	←→ (M)	↓ (C)	↑	←→	↑ (M, C)	←→ (M)	←→ (B, C)	←→ (B)	←→ (B)	←→ (B)	←→ (B)
Fractalkine/CX3CL1	↑ (B, C)	↓ (F)	↑ (M, C)	↑ 0–1 and 2–3 h	↑ (M, C)	↑ (B, C)	←→ (M) Remove undefined	←→ (B, C)	←→	←→	←→ (B, C)	←→ (M)	↓ (C)	←→ (B)	←→ (B)	←→ (B)	←→ (B)
BDNF	↑ (B, C)	↑ (C)	↑ (M, C)	NA	←→ (B, C)	↓ (C)	←→ (M, n = 1)	↓ (C)	←→	←→	↑ (F)	↑ (only M data available)	↓ (M)	←→ (B)	←→ (B)	←→ (B)	←→ (B)
NTN1	←→ (B, C)	←→ (B, C)	←→ (B, C)	←→ (logfC is 0.02 and 0.05 for 24 h and 48 h)	←→ (B, C)	←→ (B, C)	←→ (M)	↓ (B, C)	←→	←→	←→ (B, C)	←→ (M)	←→ (B, C)	←→ (B)	←→ (B)	←→ (B)	←→ (B)
PF4	↑ (F)	←→ (B, C)	↓ (M, C)	NA	←→ (B, C	←→ (B, C)	←→ (M, n = 1)	←→ (B, C)	NA	NA	←→ (B, C)	←→ (M)	↓ (F)	←→ (B)	←→ (B)	←→ (B)	←→ (B)
KL	←→ (B, C)	←→ (B, C)	←→ (B, C)	↑ 48 h	↑ (M, C)	↑ (B, C)	←→ (M) Remove undefined	←→ (B, C)	NA	←→	←→ (B, C)	↓ (M)	←→ (B, C)	NA			
FN1	←→ (B, C)	←→ (B, C)	←→ (B, C)	←→	↑ (M, C)	↑ (F, C)	←→ (M) Remove undefined	←→ (B, C)	←→	↑	←→ (B, C)	←→ (M)	↓ (C)	←→ (B)	←→ (B)	←→ (B)	←→ (B)
GPLD1	↓ (B, C)	↓ (B, C)	←→ (B, C)	↓ 0–1, 2–3, 4–6 and 48 h	←→ (B, C)	←→ (B, C)	←→ (M) Remove undefined	←→ (B, C)	↓	←→	←→ (B, C)	←→ (M)	←→ (B, C)	←→ (B)	←→ (B)	←→ (B)	←→ (B)
CLU	←→ (B, C)	←→ (B, C)	↓ (M, C)	↓ 2–3 h	←→ (B, C)	←→ (B, C)	←→ (B, C)	←→ (B, C)	NA	←→	←→ (B, C)	←→ (M)	←→ (B, C)	←→ (B)	←→ (B)	↓ (F) VL	↓ (F) VL
Fetuin-A/AHSG	←→ (M)	←→ (B, C)	←→ (B, C)	NA	←→ (B, C)	↓ (M, C)	←→ (M, n = 1)	←→ (B, C)	←→	←→	←→ (B, C)	←→ (M)	←→ (B, C)	NA			

Across the human MetaMEx, Extrameta, and MoTrPAC datasets, APLN emerged as one of the most reproducibly exercise-responsive transcripts, showing concordant upregulation in response to both acute and chronic exercise. In contrast, FNDC5 demonstrated more training-linked conservation, with shared upregulation predominantly observed under chronic exercise conditions rather than acute bouts. Notably, APLN exhibited an opposite directional response in mice under acute exercise, suggesting that while APLN is highly sensitive to exercise stimuli, its transcriptional regulation may be more dependent on species-specific physiology, timing, or experimental context. It also decreased due to inactivity in both MetaMEx’s human and mouse datasets.

In the overlap between MetaMEx (human and mouse) and Extrameta, SDC4 and MSTN represented a small set of shared directional signals spanning multiple datasets. MSTN showed consistent suppression that aligned most strongly with chronic training paradigms, consistent with its role as a negative regulator of muscle adaptation and hypertrophic remodeling. SDC4 similarly demonstrated cross-dataset concordance, suggesting that it may reflect a conserved structural or membrane-associated remodeling response to exercise that persists across experimental platforms. Importantly, the convergence of these transcripts across both species and datasets suggests that they may represent more robust components of the skeletal muscle adaptation signature than the broader set of context-specific exerkines.

Within humans, the intersection of human MetaMEx and Extrameta highlighted a larger set of consistently regulated exerkines, including METRNL, IGF1, CES2, PPARGC1A, CTSB, FSTL1, FN1, MSTN, ANGPTL4, GLPD1, and SPARC. Many of these signals are biologically coherent with known exercise-induced muscle remodeling programs, with PPARGC1A reflecting mitochondrial biogenesis and oxidative adaptation, and matrix-associated factors such as FN1 and SPARC aligning with extracellular remodeling. Importantly, this human-only concordance suggests that several exerkine-associated transcripts demonstrate reproducible responsiveness across independent human datasets, with signals variably enriched under acute exercise, more sustained under chronic training, or showing downregulation during inactivity, emphasizing that exercise modality strongly shapes the direction and persistence of transcriptional effects even within a single species. Notably, Extrameta differs methodologically from MetaMEx in that it explicitly models multiple moderators (including sex, age, training type, and time post-exercise) and applies filters to promote replicability across studies, whereas prior approaches often accounted for only a single moderator without systematically analyzing dependence on additional moderators or time-dependent gene dynamics. This difference in analytical framework likely contributes to the observed variability in exerkine transcriptional responsiveness across human datasets.

Direct cross-species overlap between MetaMEx human and mouse included GDF15, FST, DCN, TGFβ1, TGFβ2, ANGPTL4, BDNF, APLN, PPARGC1A, and SDC4, indicating that a subset of exerkine-associated transcripts exhibit conserved responsiveness across humans and rodents. However, the figure also supports that the directionality and modality dependence of these changes can diverge substantially, with several transcripts demonstrating concordant regulation in some conditions (e.g., training or inactivity) but divergent regulation in others (particularly acute exercise). This pattern is especially evident for factors such as APLN and BDNF, which appear highly exercise-responsive but may shift direction depending on acute versus chronic paradigms, tissue timing, or sampling context. Collectively, these cross-species overlaps support partial biological conservation, but also reinforce that extrapolation between humans and rodents requires careful attention to exercise modality, temporal dynamics, and the specific dataset context driving the observed signal.

Lastly, the majority of exerkines fell outside these shared regions and exhibited divergent or even opposing regulation between humans and rodent (Figure 1). Notably, many rodent-associated signals were primarily supported by MetaMEx with limited confirmation in MoTrPAC, further emphasizing the need for caution when extrapolating rodent skeletal muscle exerkine signatures to humans. Overall, acute exercise elicited broader human-specific transcriptional changes, whereas rodents demonstrated more uniform suppression patterns during inactivity, reinforcing that exerkine responsiveness cannot be inferred from a single species or exercise paradigm.

Importantly, these conclusions should be interpreted in the context of substantial cross-dataset heterogeneity, including differences in exercise modality, intensity, timing of sampling, tissue processing, cohort characteristics (e.g., age, sex, baseline fitness), and analytical pipelines across MetaMEx, Extrameta, and MoTrPAC. As a result, our cross-dataset analysis is limited by uneven study coverage, incomplete sex-stratified availability, and reliance on transcriptomic proxies that may not directly reflect secreted protein abundance or biological activity. Therefore, caution is warranted when extrapolating rodent skeletal muscle exerkine signatures to humans, and translational inferences should prioritize molecules supported across multiple datasets and species while acknowledging that divergent results may reflect both true biology and methodological variation.

## 6. Conclusions

Overall, our review (Table 1, Figure 1) suggests that APLN is the most responsive exerkine. The review also demonstrates that muscle-specific exerkine responsiveness cannot be inferred from a single species or exercise condition, and that acute vs. chronic exercise activates fundamentally different transcriptional pathways. Importantly, the strength of evidence supporting individual exerkines is variable, with some signals consistently replicated across independent datasets and platforms, while others remain context-dependent or inconsistent across studies due to differences in exercise modality, sampling time points, tissue vs. circulating measurements, and analytic pipelines. These results provide a ranked, species-informed reference for the most exercise-sensitive skeletal muscle exerkines and underscore the need for standardized, multi-species, multi-omics validation.

Moreover, because our synthesis is primarily based on transcriptomic datasets, we implicitly assume that changes in gene expression correlate with protein abundance; however, this relationship may be imperfect, and transcript-level changes do not necessarily indicate that a protein is secreted, biologically active in circulation, or taken up by distal target tissues. In the future, integrating multi-omics approaches [134] across species, tissues, and exercise paradigms will be essential to fully elucidate the complexity of skeletal muscle adaptations and to uncover the full biological and translational potential of skeletal muscle exercise-responsive exerkines.

## Data Availability

Data used in the preparation of this article were obtained from MetaMEx, Extrameta and the Molecular Transducers of Physical Activity Consortium (MoTrPAC) MoTrPACRatTraining6moData R package [version 2.0.0].
